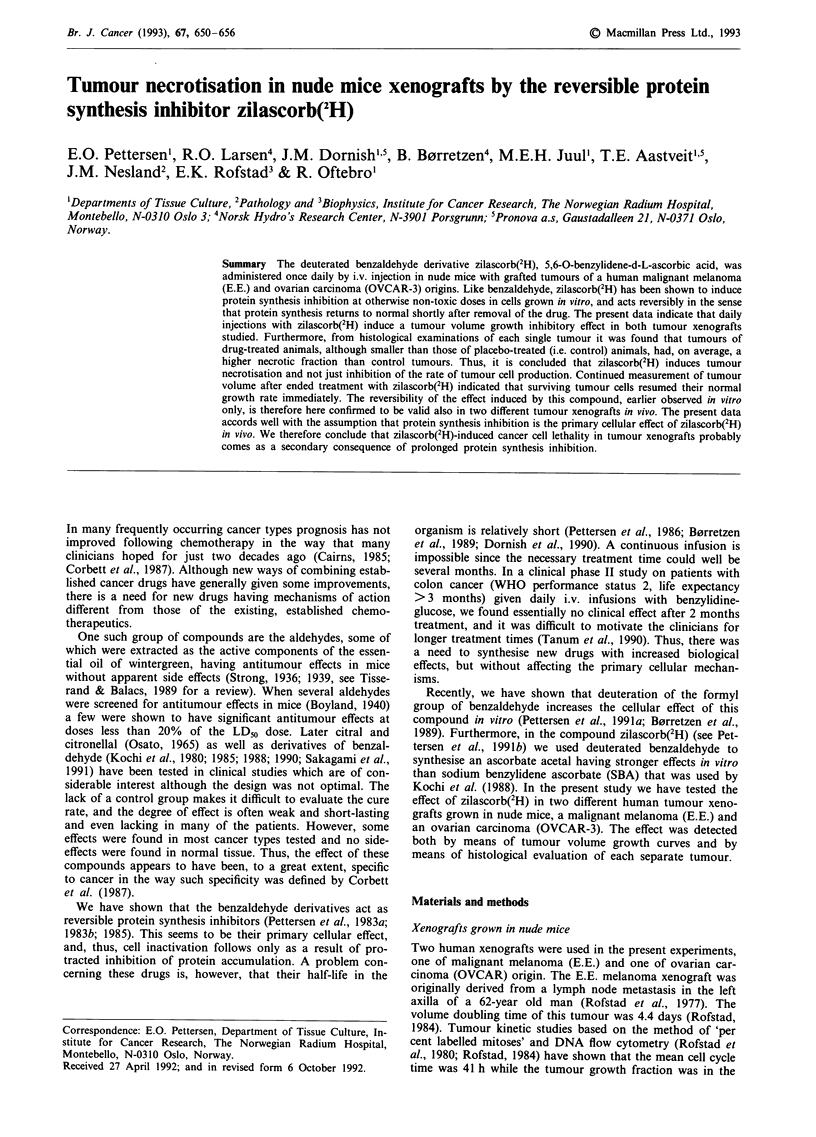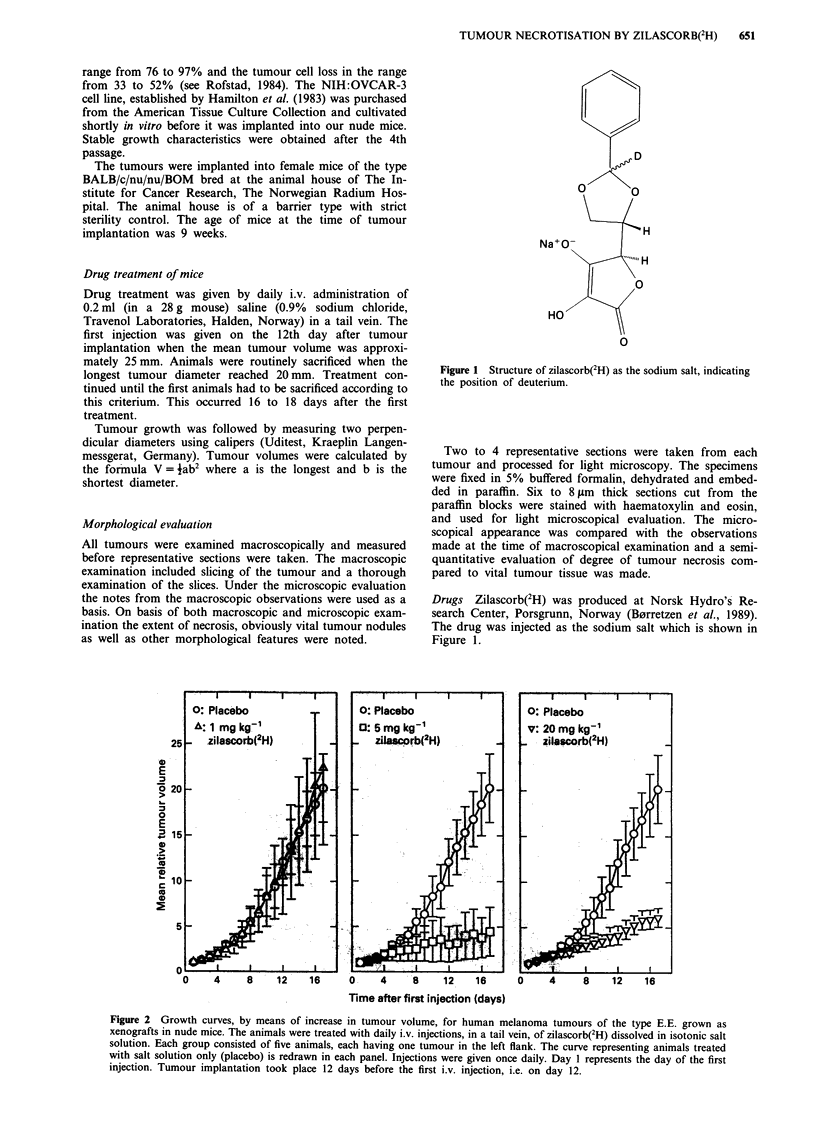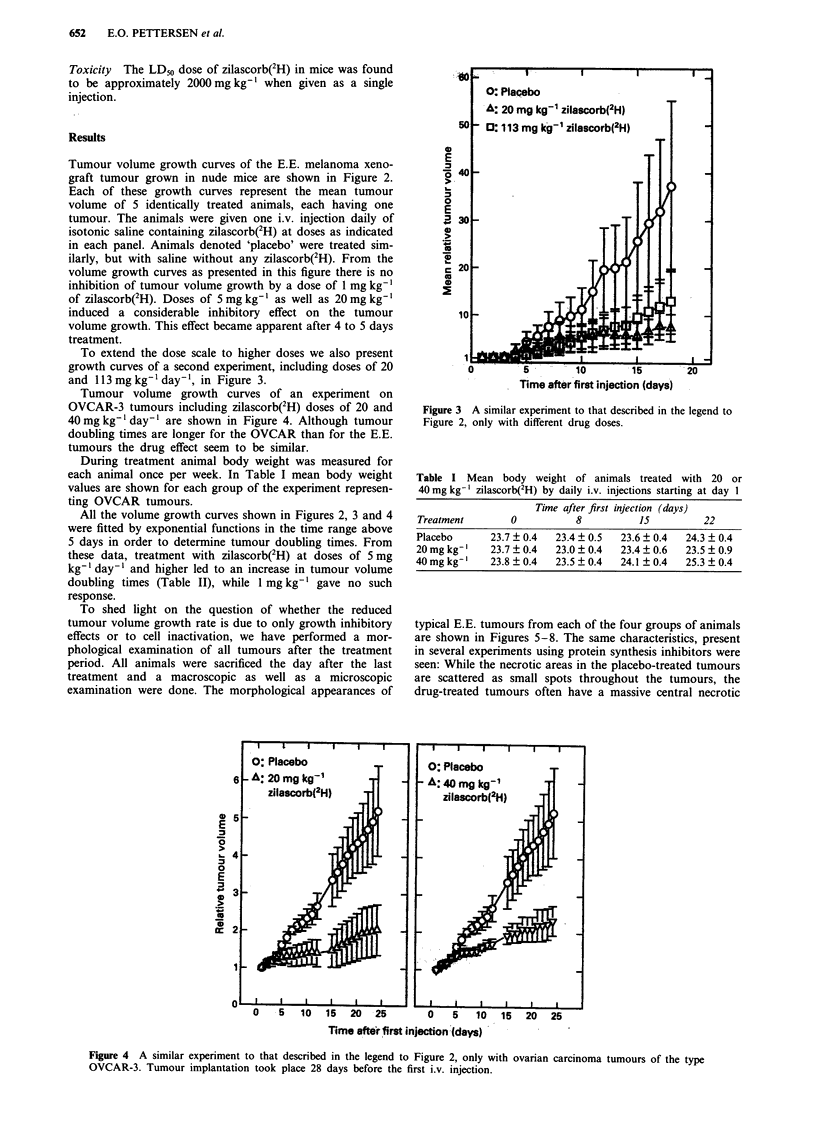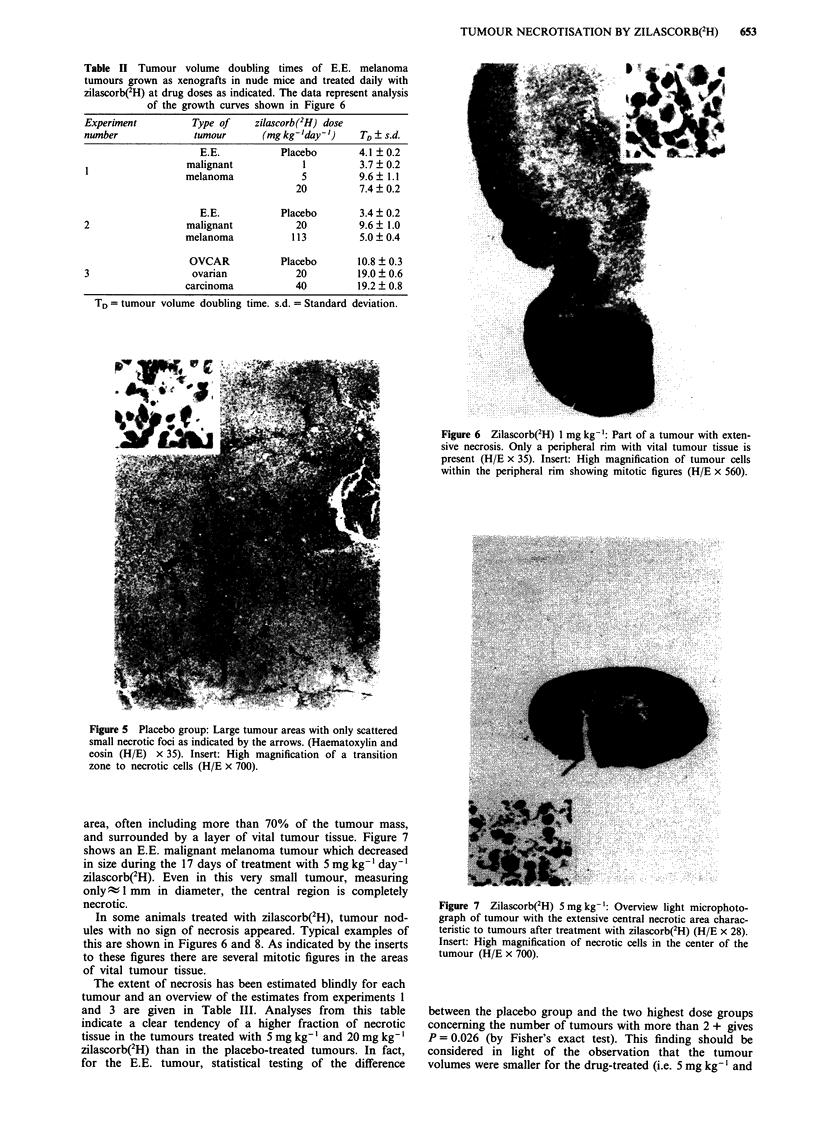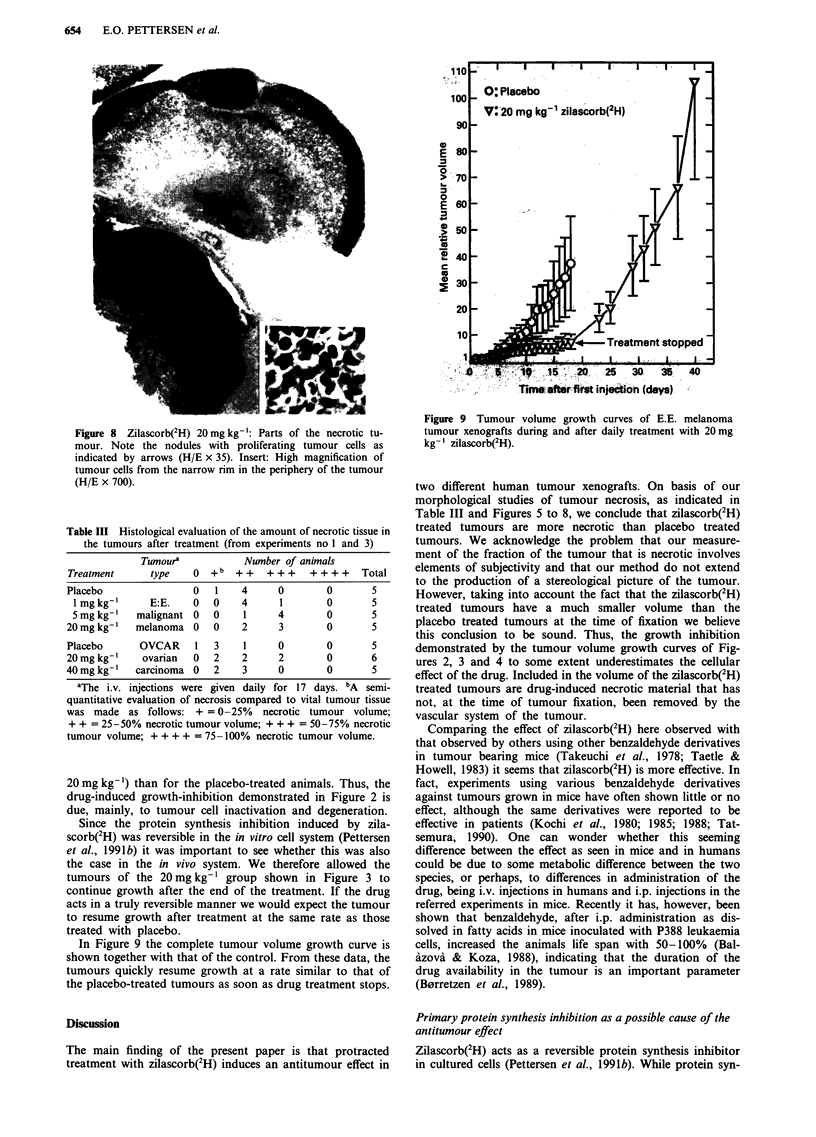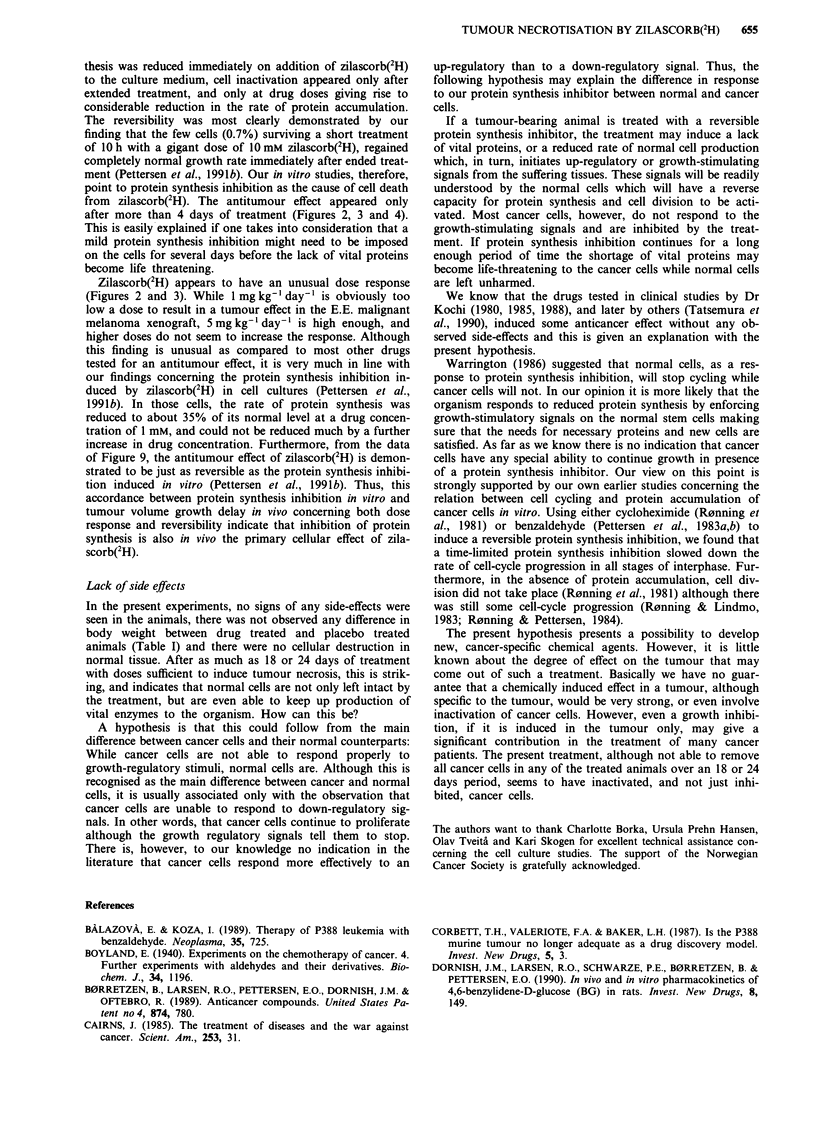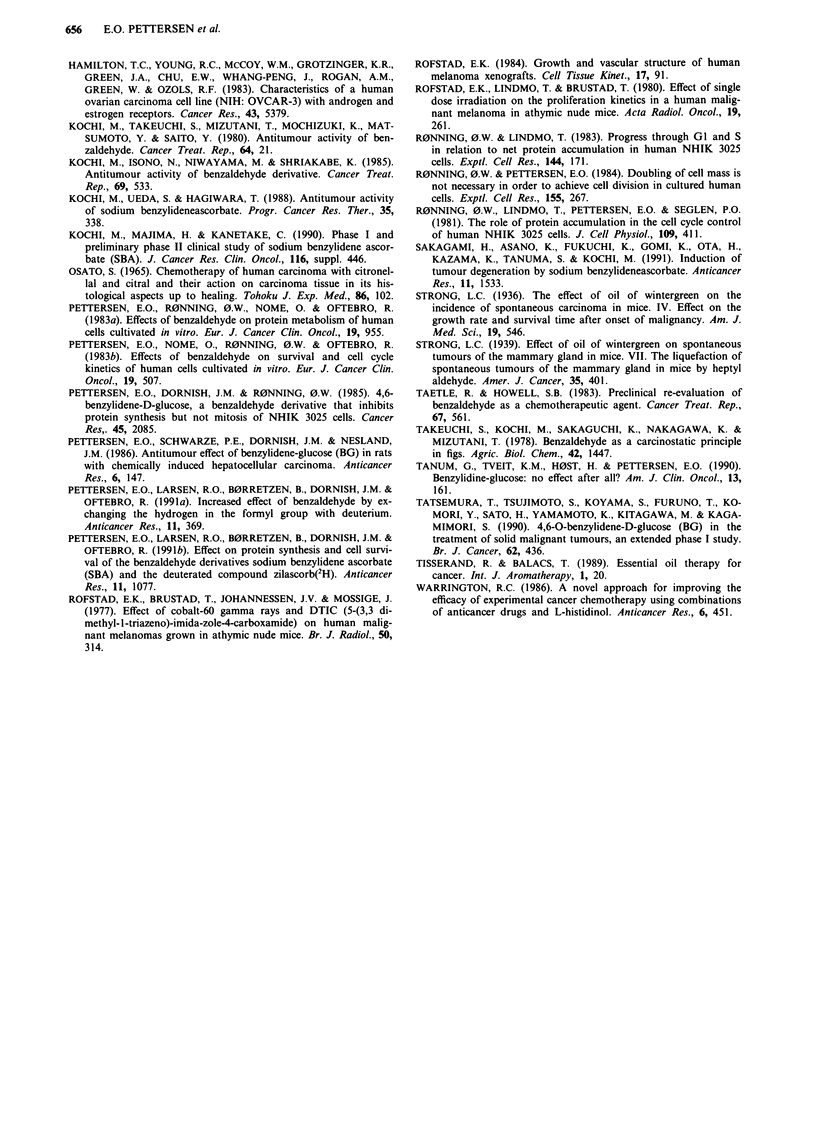# Tumour necrotisation in nude mice xenografts by the reversible protein synthesis inhibitor zilascorb(2H).

**DOI:** 10.1038/bjc.1993.121

**Published:** 1993-04

**Authors:** E. O. Pettersen, R. O. Larsen, J. M. Dornish, B. Børretzen, M. E. Juul, T. E. Aastveit, J. M. Nesland, E. K. Rofstad, R. Oftebro

**Affiliations:** Department of Tissue Culture, Norwegian Radium Hospital, Oslo.

## Abstract

**Images:**


*Br. J. Cancer* (1993), **67**, 650–656 © Macmillan Press Ltd., 1993

### Tumour necrotisation in nude mice xenografts by the reversible protein synthesis inhibitor zilascorb($^2$H)

E.O. Pettersen[1], R.O. Larsen[4], J.M. Dornish[1,5], B. Børretzen[4], M.E.H. Juul[1], T.E. Aastveit[1,5], J.M. Nesland[2], E.K. Rofstad[3] & R. Oftebro[1]

[1]*Departments of Tissue Culture,* [2]*Pathology and* [3]*Biophysics, Institute for Cancer Research, The Norwegian Radium Hospital, Montebello, N-0310 Oslo 3;* [4]*Norsk Hydro's Research Center, N-3901 Porsgrunn;* [5]*Pronova a.s, Gaustadalleen 21, N-0371 Oslo, Norway.*

**Summary** The deuterated benzaldehyde derivative zilascorb($^2$H), 5,6-O-benzylidene-d-L-ascorbic acid, was administered once daily by i.v. injection in nude mice with grafted tumours of a human malignant melanoma (E.E.) and ovarian carcinoma (OVCAR-3) origins. Like benzaldehyde, zilascorb($^2$H) has been shown to induce protein synthesis inhibition at otherwise non-toxic doses in cells grown *in vitro*, and acts reversibly in the sense that protein synthesis returns to normal shortly after removal of the drug. The present data indicate that daily injections with zilascorb($^2$H) induce a tumour volume growth inhibitory effect in both tumour xenografts studied. Furthermore, from histological examinations of each single tumour it was found that tumours of drug-treated animals, although smaller than those of placebo-treated (i.e. control) animals, had, on average, a higher necrotic fraction than control tumours. Thus, it is concluded that zilascorb($^2$H) induces tumour necrotisation and not just inhibition of the rate of tumour cell production. Continued measurement of tumour volume after ended treatment with zilascorb($^2$H) indicated that surviving tumour cells resumed their normal growth rate immediately. The reversibility of the effect induced by this compound, earlier observed *in vitro* only, is therefore here confirmed to be valid also in two different tumour xenografts *in vivo*. The present data accords well with the assumption that protein synthesis inhibition is the primary cellular effect of zilascorb($^2$H) *in vivo*. We therefore conclude that zilascorb($^2$H)-induced cancer cell lethality in tumour xenografts probably comes as a secondary consequence of prolonged protein synthesis inhibition.

In many frequently occurring cancer types prognosis has not improved following chemotherapy in the way that many clinicians hoped for just two decades ago (Cairns, 1985; Corbett *et al.*, 1987). Although new ways of combining established cancer drugs have generally given some improvements, there is a need for new drugs having mechanisms of action different from those of the existing, established chemotherapeutics.

One such group of compounds are the aldehydes, some of which were extracted as the active components of the essential oil of wintergreen, having antitumour effects in mice without apparent side effects (Strong, 1936; 1939, see Tisserand & Balacs, 1989 for a review). When several aldehydes were screened for antitumour effects in mice (Boyland, 1940) a few were shown to have significant antitumour effects at doses less than 20% of the $LD_{50}$ dose. Later citral and citronellal (Osato, 1965) as well as derivatives of benzaldehyde (Kochi *et al.*, 1980; 1985; 1988; 1990; Sakagami *et al.*, 1991) have been tested in clinical studies which are of considerable interest although the design was not optimal. The lack of a control group makes it difficult to evaluate the cure rate, and the degree of effect is often weak and short-lasting and even lacking in many of the patients. However, some effects were found in most cancer types tested and no side-effects were found in normal tissue. Thus, the effect of these compounds appears to have been, to a great extent, specific to cancer in the way such specificity was defined by Corbett *et al.* (1987).

We have shown that the benzaldehyde derivatives act as reversible protein synthesis inhibitors (Pettersen *et al.*, 1983*a*; 1983*b*; 1985). This seems to be their primary cellular effect, and, thus, cell inactivation follows only as a result of protracted inhibition of protein accumulation. A problem concerning these drugs is, however, that their half-life in the organism is relatively short (Pettersen *et al.*, 1986; Børretzen *et al.*, 1989; Dornish *et al.*, 1990). A continuous infusion is impossible since the necessary treatment time could well be several months. In a clinical phase II study on patients with colon cancer (WHO performance status 2, life expectancy >3 months) given daily i.v. infusions with benzylidine-glucose, we found essentially no clinical effect after 2 months treatment, and it was difficult to motivate the clinicians for longer treatment times (Tanum *et al.*, 1990). Thus, there was a need to synthesise new drugs with increased biological effects, but without affecting the primary cellular mechanisms.

Recently, we have shown that deuteration of the formyl group of benzaldehyde increases the cellular effect of this compound *in vitro* (Pettersen *et al.*, 1991*a*; Børretzen *et al.*, 1989). Furthermore, in the compound zilascorb($^2$H) (see Pettersen *et al.*, 1991*b*) we used deuterated benzaldehyde to synthesise an ascorbate acetal having stronger effects *in vitro* than sodium benzylidene ascorbate (SBA) that was used by Kochi *et al.* (1988). In the present study we have tested the effect of zilascorb($^2$H) in two different human tumour xenografts grown in nude mice, a malignant melanoma (E.E.) and an ovarian carcinoma (OVCAR-3). The effect was detected both by means of tumour volume growth curves and by means of histological evaluation of each separate tumour.

### Materials and methods

### *Xenografts grown in nude mice*

Two human xenografts were used in the present experiments, one of malignant melanoma (E.E.) and one of ovarian carcinoma (OVCAR) origin. The E.E. melanoma xenograft was originally derived from a lymph node metastasis in the left axilla of a 62-year old man (Rofstad *et al.*, 1977). The volume doubling time of this tumour was 4.4 days (Rofstad, 1984). Tumour kinetic studies based on the method of 'per cent labelled mitoses' and DNA flow cytometry (Rofstad *et al.*, 1980; Rofstad, 1984) have shown that the mean cell cycle time was 41 h while the tumour growth fraction was in the

Correspondence: E.O. Pettersen, Department of Tissue Culture, Institute for Cancer Research, The Norwegian Radium Hospital, Montebello, N-0310 Oslo, Norway.
Received 27 April 1992; and in revised form 6 October 1992.

range from 76 to 97% and the tumour cell loss in the range from 33 to 52% (see Rofstad, 1984). The NIH:OVCAR-3 cell line, established by Hamilton *et al.* (1983) was purchased from the American Tissue Culture Collection and cultivated shortly *in vitro* before it was implanted into our nude mice. Stable growth characteristics were obtained after the 4th passage.

The tumours were implanted into female mice of the type BALB/c/nu/nu/BOM bred at the animal house of The Institute for Cancer Research, The Norwegian Radium Hospital. The animal house is of a barrier type with strict sterility control. The age of mice at the time of tumour implantation was 9 weeks.

### *Drug treatment of mice*

Drug treatment was given by daily i.v. administration of 0.2 ml (in a 28 g mouse) saline (0.9% sodium chloride, Travenol Laboratories, Halden, Norway) in a tail vein. The first injection was given on the 12th day after tumour implantation when the mean tumour volume was approximately 25 mm. Animals were routinely sacrificed when the longest tumour diameter reached 20 mm. Treatment continued until the first animals had to be sacrificed according to this criterium. This occurred 16 to 18 days after the first treatment.

Tumour growth was followed by measuring two perpendicular diameters using calipers (Uditest, Kraeplin Langenmessgerat, Germany). Tumour volumes were calculated by the formula $V = \frac{1}{2}ab^2$ where a is the longest and b is the shortest diameter.

### *Morphological evaluation*

All tumours were examined macroscopically and measured before representative sections were taken. The macroscopic examination included slicing of the tumour and a thorough examination of the slices. Under the microscopic evaluation the notes from the macroscopic observations were used as a basis. On basis of both macroscopic and microscopic examination the extent of necrosis, obviously vital tumour nodules as well as other morphological features were noted.

**Figure 1** Structure of zilascorb($^2$H) as the sodium salt, indicating the position of deuterium.

Two to 4 representative sections were taken from each tumour and processed for light microscopy. The specimens were fixed in 5% buffered formalin, dehydrated and embedded in paraffin. Six to 8 μm thick sections cut from the paraffin blocks were stained with haematoxylin and eosin, and used for light microscopical evaluation. The microscopical appearance was compared with the observations made at the time of macroscopical examination and a semiquantitative evaluation of degree of tumour necrosis compared to vital tumour tissue was made.

*Drugs* Zilascorb($^2$H) was produced at Norsk Hydro's Research Center, Porsgrunn, Norway (Børretzen *et al.*, 1989). The drug was injected as the sodium salt which is shown in Figure 1.

**Figure 2** Growth curves, by means of increase in tumour volume, for human melanoma tumours of the type E.E. grown as xenografts in nude mice. The animals were treated with daily i.v. injections, in a tail vein, of zilascorb($^2$H) dissolved in isotonic salt solution. Each group consisted of five animals, each having one tumour in the left flank. The curve representing animals treated with salt solution only (placebo) is redrawn in each panel. Injections were given once daily. Day 1 represents the day of the first injection. Tumour implantation took place 12 days before the first i.v. injection, i.e. on day 12.

*Toxicity* The $LD_{50}$ dose of zilascorb($^2$H) in mice was found to be approximately 2000 mg kg$^{-1}$ when given as a single injection.

### Results

Tumour volume growth curves of the E.E. melanoma xenograft tumour grown in nude mice are shown in Figure 2. Each of these growth curves represent the mean tumour volume of 5 identically treated animals, each having one tumour. The animals were given one i.v. injection daily of isotonic saline containing zilascorb($^2$H) at doses as indicated in each panel. Animals denoted 'placebo' were treated similarly, but with saline without any zilascorb($^2$H). From the volume growth curves as presented in this figure there is no inhibition of tumour volume growth by a dose of 1 mg kg$^{-1}$ of zilascorb($^2$H). Doses of 5 mg kg$^{-1}$ as well as 20 mg kg$^{-1}$ induced a considerable inhibitory effect on the tumour volume growth. This effect became apparent after 4 to 5 days treatment.

To extend the dose scale to higher doses we also present growth curves of a second experiment, including doses of 20 and 113 mg kg$^{-1}$ day$^{-1}$, in Figure 3.

Tumour volume growth curves of an experiment on OVCAR-3 tumours including zilascorb($^2$H) doses of 20 and 40 mg kg$^{-1}$ day$^{-1}$ are shown in Figure 4. Although tumour doubling times are longer for the OVCAR than for the E.E. tumours the drug effect seem to be similar.

During treatment animal body weight was measured for each animal once per week. In Table I mean body weight values are shown for each group of the experiment representing OVCAR tumours.

All the volume growth curves shown in Figures 2, 3 and 4 were fitted by exponential functions in the time range above 5 days in order to determine tumour doubling times. From these data, treatment with zilascorb($^2$H) at doses of 5 mg kg$^{-1}$ day$^{-1}$ and higher led to an increase in tumour volume doubling times (Table II), while 1 mg kg$^{-1}$ gave no such response.

To shed light on the question of whether the reduced tumour volume growth rate is due to only growth inhibitory effects or to cell inactivation, we have performed a morphological examination of all tumours after the treatment period. All animals were sacrificed the day after the last treatment and a macroscopic as well as a microscopic examination were done. The morphological appearances of typical E.E. tumours from each of the four groups of animals are shown in Figures 5–8. The same characteristics, present in several experiments using protein synthesis inhibitors were seen: While the necrotic areas in the placebo-treated tumours are scattered as small spots throughout the tumours, the drug-treated tumours often have a massive central necrotic

**Figure 3** A similar experiment to that described in the legend to Figure 2, only with different drug doses.

**Table I** Mean body weight of animals treated with 20 or 40 mg kg$^{-1}$ zilascorb($^2$H) by daily i.v. injections starting at day 1

| Treatment | *Time after first injection (days)* 0 | 8 | 15 | 22 |
|---|---|---|---|---|
| Placebo | 23.7 ± 0.4 | 23.4 ± 0.5 | 23.6 ± 0.4 | 24.3 ± 0.4 |
| 20 mg kg$^{-1}$ | 23.7 ± 0.4 | 23.0 ± 0.4 | 23.4 ± 0.6 | 23.5 ± 0.9 |
| 40 mg kg$^{-1}$ | 23.8 ± 0.4 | 23.5 ± 0.4 | 24.1 ± 0.4 | 25.3 ± 0.4 |

**Figure 4** A similar experiment to that described in the legend to Figure 2, only with ovarian carcinoma tumours of the type OVCAR-3. Tumour implantation took place 28 days before the first i.v. injection.

**Table II** Tumour volume doubling times of E.E. melanoma tumours grown as xenografts in nude mice and treated daily with zilascorb($^2$H) at drug doses as indicated. The data represent analysis of the growth curves shown in Figure 6

| *Experiment number* | *Type of tumour* | *zilascorb($^2$H) dose ($mg\,kg^{-1}day^{-1}$)* | $T_D \pm$ *s.d.* |
|---|---|---|---|
| | E.E. | Placebo | 4.1 ± 0.2 |
| 1 | malignant | 1 | 3.7 ± 0.2 |
| | melanoma | 5 | 9.6 ± 1.1 |
| | | 20 | 7.4 ± 0.2 |
| | E.E. | Placebo | 3.4 ± 0.2 |
| 2 | malignant | 20 | 9.6 ± 1.0 |
| | melanoma | 113 | 5.0 ± 0.4 |
| | OVCAR | Placebo | 10.8 ± 0.3 |
| 3 | ovarian | 20 | 19.0 ± 0.6 |
| | carcinoma | 40 | 19.2 ± 0.8 |

$T_D$ = tumour volume doubling time. s.d. = Standard deviation.

**Figure 5** Placebo group: Large tumour areas with only scattered small necrotic foci as indicated by the arrows. (Haematoxylin and eosin (H/E) × 35). Insert: High magnification of a transition zone to necrotic cells (H/E × 700).

area, often including more than 70% of the tumour mass, and surrounded by a layer of vital tumour tissue. Figure 7 shows an E.E. malignant melanoma tumour which decreased in size during the 17 days of treatment with 5 mg kg$^{-1}$ day$^{-1}$ zilascorb($^2$H). Even in this very small tumour, measuring only ≈ 1 mm in diameter, the central region is completely necrotic.

In some animals treated with zilascorb($^2$H), tumour nodules with no sign of necrosis appeared. Typical examples of this are shown in Figures 6 and 8. As indicated by the inserts to these figures there are several mitotic figures in the areas of vital tumour tissue.

The extent of necrosis has been estimated blindly for each tumour and an overview of the estimates from experiments 1 and 3 are given in Table III. Analyses from this table indicate a clear tendency of a higher fraction of necrotic tissue in the tumours treated with 5 mg kg$^{-1}$ and 20 mg kg$^{-1}$ zilascorb($^2$H) than in the placebo-treated tumours. In fact, for the E.E. tumour, statistical testing of the difference

**Figure 6** Zilascorb($^2$H) 1 mg kg$^{-1}$: Part of a tumour with extensive necrosis. Only a peripheral rim with vital tumour tissue is present (H/E × 35). Insert: High magnification of tumour cells within the peripheral rim showing mitotic figures (H/E × 560).

**Figure 7** Zilascorb($^2$H) 5 mg kg$^{-1}$: Overview light microphotograph of tumour with the extensive central necrotic area characteristic to tumours after treatment with zilascorb($^2$H) (H/E × 28). Insert: High magnification of necrotic cells in the center of the tumour (H/E × 700).

between the placebo group and the two highest dose groups concerning the number of tumours with more than 2 + gives $P = 0.026$ (by Fisher's exact test). This finding should be considered in light of the observation that the tumour volumes were smaller for the drug-treated (i.e. 5 mg kg$^{-1}$ and

**Figure 8** Zilascorb($^2$H) 20 mg kg$^{-1}$: Parts of the necrotic tumour. Note the nodules with proliferating tumour cells as indicated by arrows (H/E × 35). Insert: High magnification of tumour cells from the narrow rim in the periphery of the tumour (H/E × 700).

**Figure 9** Tumour volume growth curves of E.E. melanoma tumour xenografts during and after daily treatment with 20 mg kg$^{-1}$ zilascorb($^2$H).

**Table III** Histological evaluation of the amount of necrotic tissue in the tumours after treatment (from experiments no 1 and 3)

| Treatment | Tumour[a] type | Number of animals 0 | +[b] | ++ | +++ | ++++ | Total |
|---|---|---|---|---|---|---|---|
| Placebo | | 0 | 1 | 4 | 0 | 0 | 5 |
| 1 mg kg$^{-1}$ | E:E. | 0 | 0 | 4 | 1 | 0 | 5 |
| 5 mg kg$^{-1}$ | malignant | 0 | 0 | 1 | 4 | 0 | 5 |
| 20 mg kg$^{-1}$ | melanoma | 0 | 0 | 2 | 3 | 0 | 5 |
| Placebo | OVCAR | 1 | 3 | 1 | 0 | 0 | 5 |
| 20 mg kg$^{-1}$ | ovarian | 0 | 2 | 2 | 2 | 0 | 6 |
| 40 mg kg$^{-1}$ | carcinoma | 0 | 2 | 3 | 0 | 0 | 5 |

[a]The i.v. injections were given daily for 17 days. [b]A semi-quantitative evaluation of necrosis compared to vital tumour tissue was made as follows: + = 0–25% necrotic tumour volume; + + = 25–50% necrotic tumour volume; + + + = 50–75% necrotic tumour volume; + + + + = 75–100% necrotic tumour volume.

20 mg kg$^{-1}$) than for the placebo-treated animals. Thus, the drug-induced growth-inhibition demonstrated in Figure 2 is due, mainly, to tumour cell inactivation and degeneration.

Since the protein synthesis inhibition induced by zilascorb($^2$H) was reversible in the *in vitro* cell system (Pettersen *et al.*, 1991*b*) it was important to see whether this was also the case in the *in vivo* system. We therefore allowed the tumours of the 20 mg kg$^{-1}$ group shown in Figure 3 to continue growth after the end of the treatment. If the drug acts in a truly reversible manner we would expect the tumour to resume growth after treatment at the same rate as those treated with placebo.

In Figure 9 the complete tumour volume growth curve is shown together with that of the control. From these data, the tumours quickly resume growth at a rate similar to that of the placebo-treated tumours as soon as drug treatment stops.

### Discussion

The main finding of the present paper is that protracted treatment with zilascorb($^2$H) induces an antitumour effect in two different human tumour xenografts. On basis of our morphological studies of tumour necrosis, as indicated in Table III and Figures 5 to 8, we conclude that zilascorb($^2$H) treated tumours are more necrotic than placebo treated tumours. We acknowledge the problem that our measurement of the fraction of the tumour that is necrotic involves elements of subjectivity and that our method do not extend to the production of a stereological picture of the tumour. However, taking into account the fact that the zilascorb($^2$H) treated tumours have a much smaller volume than the placebo treated tumours at the time of fixation we believe this conclusion to be sound. Thus, the growth inhibition demonstrated by the tumour volume growth curves of Figures 2, 3 and 4 to some extent underestimates the cellular effect of the drug. Included in the volume of the zilascorb($^2$H) treated tumours are drug-induced necrotic material that has not, at the time of tumour fixation, been removed by the vascular system of the tumour.

Comparing the effect of zilascorb($^2$H) here observed with that observed by others using other benzaldehyde derivatives in tumour bearing mice (Takeuchi *et al.*, 1978; Taetle & Howell, 1983) it seems that zilascorb($^2$H) is more effective. In fact, experiments using various benzaldehyde derivatives against tumours grown in mice have often shown little or no effect, although the same derivatives were reported to be effective in patients (Kochi *et al.*, 1980; 1985; 1988; Tatsemura, 1990). One can wonder whether this seeming difference between the effect as seen in mice and in humans could be due to some metabolic difference between the two species, or perhaps, to differences in administration of the drug, being i.v. injections in humans and i.p. injections in the referred experiments in mice. Recently it has, however, been shown that benzaldehyde, after i.p. administration as dissolved in fatty acids in mice inoculated with P388 leukaemia cells, increased the animals life span with 50–100% (Balàzovà & Koza, 1988), indicating that the duration of the drug availability in the tumour is an important parameter (Børretzen *et al.*, 1989).

### *Primary protein synthesis inhibition as a possible cause of the antitumour effect*

Zilascorb($^2$H) acts as a reversible protein synthesis inhibitor in cultured cells (Pettersen *et al.*, 1991*b*). While protein synthesis was reduced immediately on addition of zilascorb($^2$H) to the culture medium, cell inactivation appeared only after extended treatment, and only at drug doses giving rise to considerable reduction in the rate of protein accumulation. The reversibility was most clearly demonstrated by our finding that the few cells (0.7%) surviving a short treatment of 10 h with a gigant dose of 10 mM zilascorb($^2$H), regained completely normal growth rate immediately after ended treatment (Pettersen *et al.*, 1991*b*). Our *in vitro* studies, therefore, point to protein synthesis inhibition as the cause of cell death from zilascorb($^2$H). The antitumour effect appeared only after more than 4 days of treatment (Figures 2, 3 and 4). This is easily explained if one takes into consideration that a mild protein synthesis inhibition might need to be imposed on the cells for several days before the lack of vital proteins become life threatening.

Zilascorb($^2$H) appears to have an unusual dose response (Figures 2 and 3). While 1 mg kg$^{-1}$ day$^{-1}$ is obviously too low a dose to result in a tumour effect in the E.E. malignant melanoma xenograft, 5 mg kg$^{-1}$ day$^{-1}$ is high enough, and higher doses do not seem to increase the response. Although this finding is unusual as compared to most other drugs tested for an antitumour effect, it is very much in line with our findings concerning the protein synthesis inhibition induced by zilascorb($^2$H) in cell cultures (Pettersen *et al.*, 1991*b*). In those cells, the rate of protein synthesis was reduced to about 35% of its normal level at a drug concentration of 1 mM, and could not be reduced much by a further increase in drug concentration. Furthermore, from the data of Figure 9, the antitumour effect of zilascorb($^2$H) is demonstrated to be just as reversible as the protein synthesis inhibition induced *in vitro* (Pettersen *et al.*, 1991*b*). Thus, this accordance between protein synthesis inhibition *in vitro* and tumour volume growth delay *in vivo* concerning both dose response and reversibility indicate that inhibition of protein synthesis is also *in vivo* the primary cellular effect of zilascorb($^2$H).

### *Lack of side effects*

In the present experiments, no signs of any side-effects were seen in the animals, there was not observed any difference in body weight between drug treated and placebo treated animals (Table I) and there were no cellular destruction in normal tissue. After as much as 18 or 24 days of treatment with doses sufficient to induce tumour necrosis, this is striking, and indicates that normal cells are not only left intact by the treatment, but are even able to keep up production of vital enzymes to the organism. How can this be?

A hypothesis is that this could follow from the main difference between cancer cells and their normal counterparts: While cancer cells are not able to respond properly to growth-regulatory stimuli, normal cells are. Although this is recognised as the main difference between cancer and normal cells, it is usually associated only with the observation that cancer cells are unable to respond to down-regulatory signals. In other words, that cancer cells continue to proliferate although the growth regulatory signals tell them to stop. There is, however, to our knowledge no indication in the literature that cancer cells respond more effectively to an up-regulatory than to a down-regulatory signal. Thus, the following hypothesis may explain the difference in response to our protein synthesis inhibitor between normal and cancer cells.

If a tumour-bearing animal is treated with a reversible protein synthesis inhibitor, the treatment may induce a lack of vital proteins, or a reduced rate of normal cell production which, in turn, initiates up-regulatory or growth-stimulating signals from the suffering tissues. These signals will be readily understood by the normal cells which will have a reverse capacity for protein synthesis and cell division to be activated. Most cancer cells, however, do not respond to the growth-stimulating signals and are inhibited by the treatment. If protein synthesis inhibition continues for a long enough period of time the shortage of vital proteins may become life-threatening to the cancer cells while normal cells are left unharmed.

We know that the drugs tested in clinical studies by Dr Kochi (1980, 1985, 1988), and later by others (Tatsemura *et al.*, 1990), induced some anticancer effect without any observed side-effects and this is given an explanation with the present hypothesis.

Warrington (1986) suggested that normal cells, as a response to protein synthesis inhibition, will stop cycling while cancer cells will not. In our opinion it is more likely that the organism responds to reduced protein synthesis by enforcing growth-stimulatory signals on the normal stem cells making sure that the needs for necessary proteins and new cells are satisfied. As far as we know there is no indication that cancer cells have any special ability to continue growth in presence of a protein synthesis inhibitor. Our view on this point is strongly supported by our own earlier studies concerning the relation between cell cycling and protein accumulation of cancer cells *in vitro*. Using either cycloheximide (Rønning *et al.*, 1981) or benzaldehyde (Pettersen *et al.*, 1983*a*,*b*) to induce a reversible protein synthesis inhibition, we found that a time-limited protein synthesis inhibition slowed down the rate of cell-cycle progression in all stages of interphase. Furthermore, in the absence of protein accumulation, cell division did not take place (Rønning *et al.*, 1981) although there was still some cell-cycle progression (Rønning & Lindmo, 1983; Rønning & Pettersen, 1984).

The present hypothesis presents a possibility to develop new, cancer-specific chemical agents. However, it is little known about the degree of effect on the tumour that may come out of such a treatment. Basically we have no guarantee that a chemically induced effect in a tumour, although specific to the tumour, would be very strong, or even involve inactivation of cancer cells. However, even a growth inhibition, if it is induced in the tumour only, may give a significant contribution in the treatment of many cancer patients. The present treatment, although not able to remove all cancer cells in any of the treated animals over an 18 or 24 days period, seems to have inactivated, and not just inhibited, cancer cells.

The authors want to thank Charlotte Borka, Ursula Prehn Hansen, Olav Tveitå and Kari Skogen for excellent technical assistance concerning the cell culture studies. The support of the Norwegian Cancer Society is gratefully acknowledged.

### References

BÅLAZOVÅ, E. & KOZA, I. (1989). Therapy of P388 leukemia with benzaldehyde. *Neoplasma*, **35**, 725.

BOYLAND, E. (1940). Experiments on the chemotherapy of cancer. 4. Further experiments with aldehydes and their derivatives. *Biochem. J.*, **34**, 1196.

BØRRETZEN, B., LARSEN, R.O., PETTERSEN, E.O., DORNISH, J.M. & OFTEBRO, R. (1989). Anticancer compounds. *United States Patent no 4*, **874**, 780.

CAIRNS, J. (1985). The treatment of diseases and the war against cancer. *Scient. Am.*, **253**, 31.

CORBETT, T.H., VALERIOTE, F.A. & BAKER, L.H. (1987). Is the P388 murine tumour no longer adequate as a drug discovery model. *Invest. New Drugs*, **5**, 3.

DORNISH, J.M., LARSEN, R.O., SCHWARZE, P.E., BØRRETZEN, B. & PETTERSEN, E.O. (1990). *In vivo* and *in vitro* pharmacokinetics of 4,6-benzylidene-D-glucose (BG) in rats. *Invest. New Drugs*, **8**, 149.

HAMILTON, T.C., YOUNG, R.C., McCOY, W.M., GROTZINGER, K.R., GREEN, J.A., CHU, E.W., WHANG-PENG, J., ROGAN, A.M., GREEN, W. & OZOLS, R.F. (1983). Characteristics of a human ovarian carcinoma cell line (NIH: OVCAR-3) with androgen and estrogen receptors. *Cancer Res.*, **43**, 5379.

KOCHI, M., TAKEUCHI, S., MIZUTANI, T., MOCHIZUKI, K., MATSUMOTO, Y. & SAITO, Y. (1980). Antitumour activity of benzaldehyde. *Cancer Treat. Rep.*, **64**, 21.

KOCHI, M., ISONO, N., NIWAYAMA, M. & SHRIAKABE, K. (1985). Antitumour activity of benzaldehyde derivative. *Cancer Treat. Rep.*, **69**, 533.

KOCHI, M., UEDA, S. & HAGIWARA, T. (1988). Antitumour activity of sodium benzylideneascorbate. *Progr. Cancer Res. Ther.*, **35**, 338.

KOCHI, M., MAJIMA, H. & KANETAKE, C. (1990). Phase I and preliminary phase II clinical study of sodium benzylidene ascorbate (SBA). *J. Cancer Res. Clin. Oncol.*, **116**, suppl. 446.

OSATO, S. (1965). Chemotherapy of human carcinoma with citronellal and citral and their action on carcinoma tissue in its histological aspects up to healing. *Tohoku J. Exp. Med.*, **86**, 102.

PETTERSEN, E.O., RØNNING, Ø.W., NOME, O. & OFTEBRO, R. (1983*a*). Effects of benzaldehyde on protein metabolism of human cells cultivated *in vitro*. *Eur. J. Cancer Clin. Oncol.*, **19**, 955.

PETTERSEN, E.O., NOME, O., RØNNING, Ø.W. & OFTEBRO, R. (1983*b*). Effects of benzaldehyde on survival and cell cycle kinetics of human cells cultivated *in vitro*. *Eur. J. Cancer Clin. Oncol.*, **19**, 507.

PETTERSEN, E.O., DORNISH, J.M. & RØNNING, Ø.W. (1985). 4,6-benzylidene-D-glucose, a benzaldehyde derivative that inhibits protein synthesis but not mitosis of NHIK 3025 cells. *Cancer Res*,. **45**, 2085.

PETTERSEN, E.O., SCHWARZE, P.E., DORNISH, J.M. & NESLAND, J.M. (1986). Antitumour effect of benzylidene-glucose (BG) in rats with chemically induced hepatocellular carcinoma. *Anticancer Res.*, **6**, 147.

PETTERSEN, E.O., LARSEN, R.O., BØRRETZEN, B., DORNISH, J.M. & OFTEBRO, R. (1991*a*). Increased effect of benzaldehyde by exchanging the hydrogen in the formyl group with deuterium. *Anticancer Res.*, **11**, 369.

PETTERSEN, E.O., LARSEN, R.O., BØRRETZEN, B., DORNISH, J.M. & OFTEBRO, R. (1991*b*). Effect on protein synthesis and cell survival of the benzaldehyde derivatives sodium benzylidene ascorbate (SBA) and the deuterated compound zilascorb($^2$H). *Anticancer Res.*, **11**, 1077.

ROFSTAD, E.K., BRUSTAD, T., JOHANNESSEN, J.V. & MOSSIGE, J. (1977). Effect of cobalt-60 gamma rays and DTIC (5-(3,3 dimethyl-1-triazeno)-imida-zole-4-carboxamide) on human malignant melanomas grown in athymic nude mice. *Br. J. Radiol.*, **50**, 314.

ROFSTAD, E.K. (1984). Growth and vascular structure of human melanoma xenografts. *Cell Tissue Kinet.*, **17**, 91.

ROFSTAD, E.K., LINDMO, T. & BRUSTAD, T. (1980). Effect of single dose irradiation on the proliferation kinetics in a human malignant melanoma in athymic nude mice. *Acta Radiol. Oncol.*, **19**, 261.

RØNNING, Ø.W. & LINDMO, T. (1983). Progress through G1 and S in relation to net protein accumulation in human NHIK 3025 cells. *Exptl. Cell Res.*, **144**, 171.

RØNNING, Ø.W. & PETTERSEN, E.O. (1984). Doubling of cell mass is not necessary in order to achieve cell division in cultured human cells. *Exptl. Cell Res.*, **155**, 267.

RØNNING, Ø.W., LINDMO, T., PETTERSEN, E.O. & SEGLEN, P.O. (1981). The role of protein accumulation in the cell cycle control of human NHIK 3025 cells. *J. Cell Physiol.*, **109**, 411.

SAKAGAMI, H., ASANO, K., FUKUCHI, K., GOMI, K., OTA, H., KAZAMA, K., TANUMA, S. & KOCHI, M. (1991). Induction of tumour degeneration by sodium benzylideneascorbate. *Anticancer Res.*, **11**, 1533.

STRONG, L.C. (1936). The effect of oil of wintergreen on the incidence of spontaneous carcinoma in mice. IV. Effect on the growth rate and survival time after onset of malignancy. *Am. J. Med. Sci.*, **19**, 546.

STRONG, L.C. (1939). Effect of oil of wintergreen on spontaneous tumours of the mammary gland in mice. VII. The liquefaction of spontaneous tumours of the mammary gland in mice by heptyl aldehyde. *Amer. J. Cancer*, **35**, 401.

TAETLE, R. & HOWELL, S.B. (1983). Preclinical re-evaluation of benzaldehyde as a chemotherapeutic agent. *Cancer Treat. Rep.*, **67**, 561.

TAKEUCHI, S., KOCHI, M., SAKAGUCHI, K., NAKAGAWA, K. & MIZUTANI, T. (1978). Benzaldehyde as a carcinostatic principle in figs. *Agric. Biol. Chem.*, **42**, 1447.

TANUM, G., TVEIT, K.M., HØST, H. & PETTERSEN, E.O. (1990). Benzylidine-glucose: no effect after all? *Am. J. Clin. Oncol.*, **13**, 161.

TATSEMURA, T., TSUJIMOTO, S., KOYAMA, S., FURUNO, T., KOMORI, Y., SATO, H., YAMAMOTO, K., KITAGAWA, M. & KAGAMIMORI, S. (1990). 4,6-O-benzylidene-D-glucose (BG) in the treatment of solid malignant tumours, an extended phase I study. *Br. J. Cancer*, **62**, 436.

TISSERAND, R. & BALACS, T. (1989). Essential oil therapy for cancer. *Int. J. Aromatherapy*, **1**, 20.

WARRINGTON, R.C. (1986). A novel approach for improving the efficacy of experimental cancer chemotherapy using combinations of anticancer drugs and L-histidinol. *Anticancer Res.*, **6**, 451.